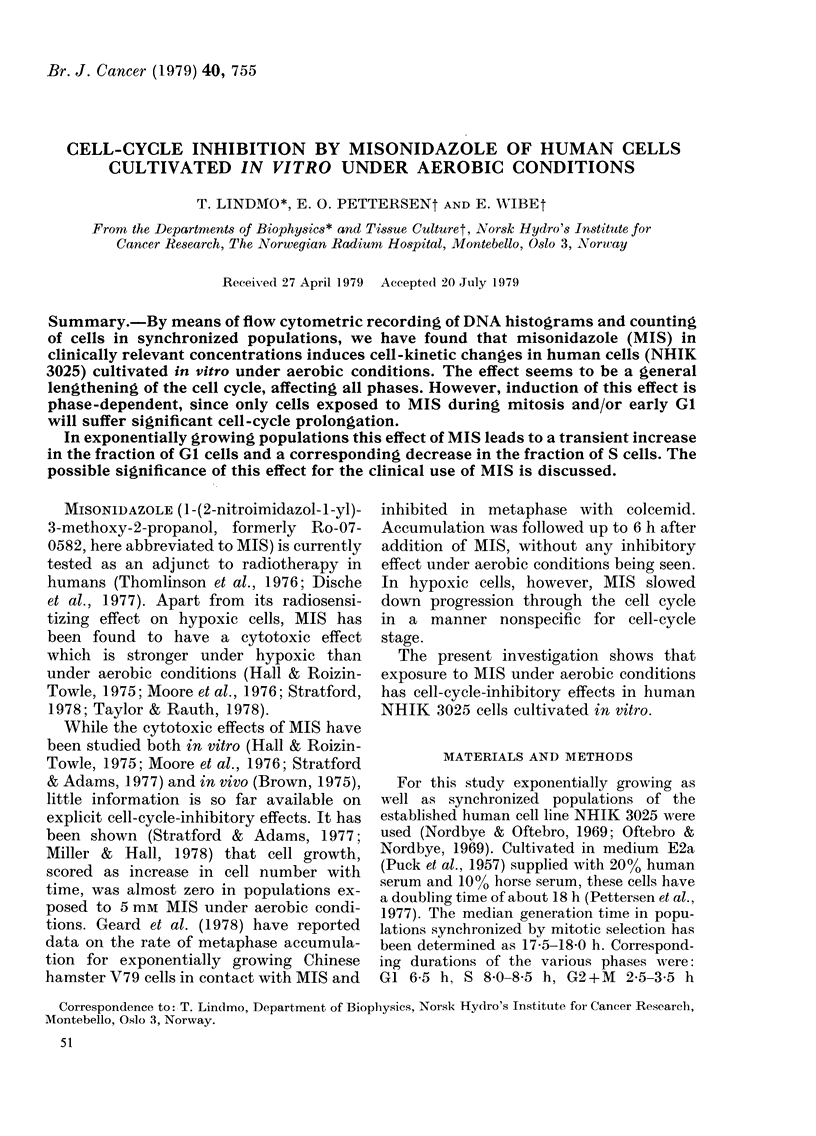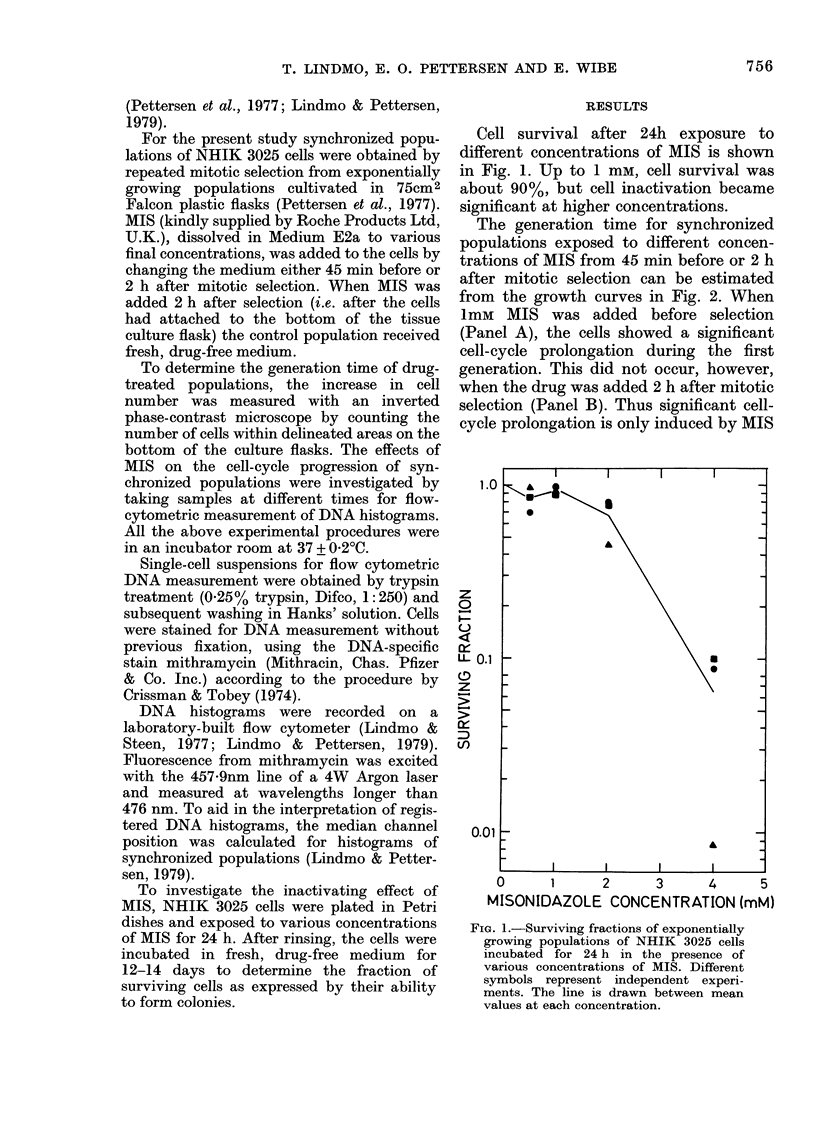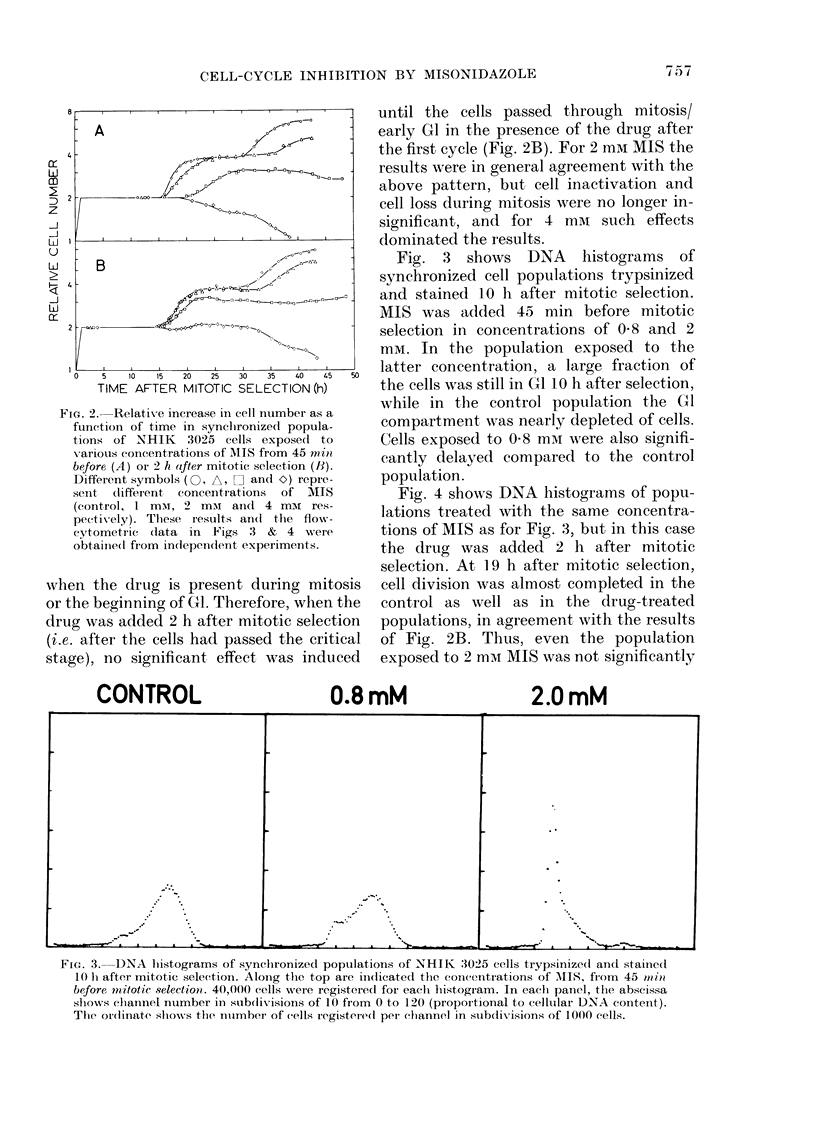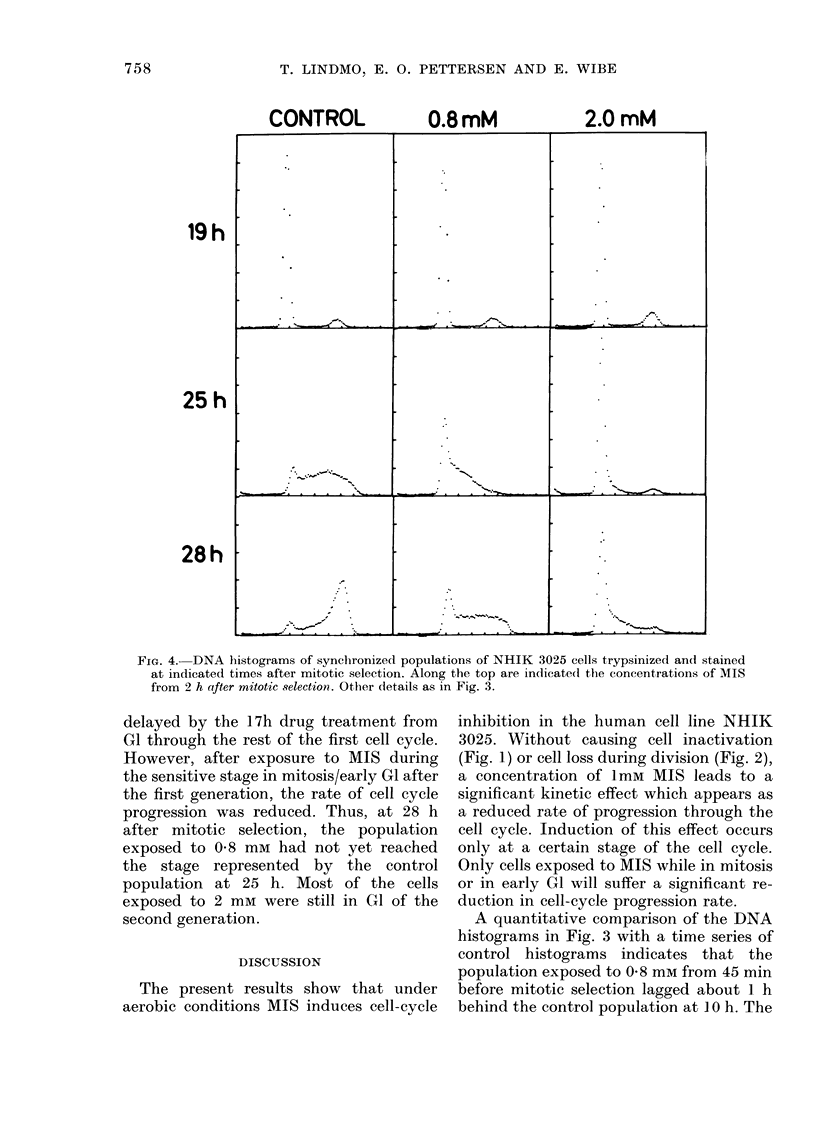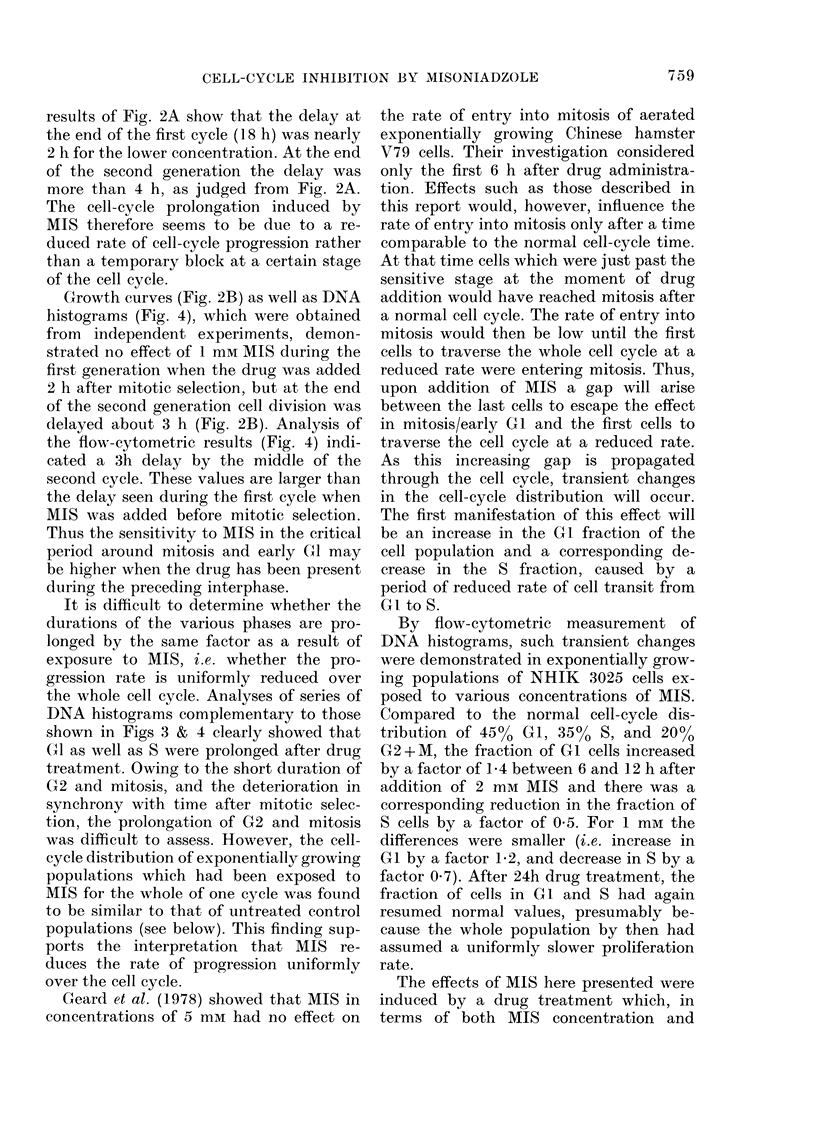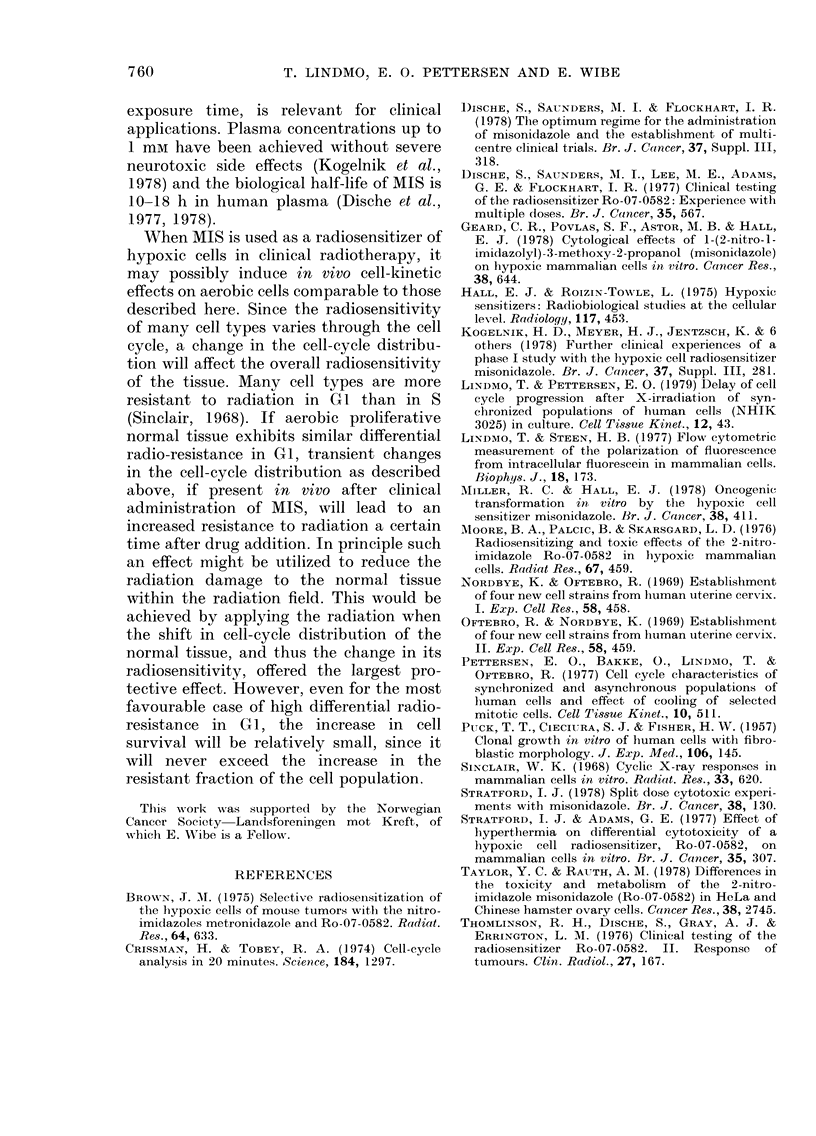# Cell-cycle inhibition by misonidazole of human cells cultivated in vitro under aerobic conditions.

**DOI:** 10.1038/bjc.1979.257

**Published:** 1979-11

**Authors:** T. Lindmo, E. O. Pettersen, E. Wibe

## Abstract

By means of flow cytometric recording of DNA histograms and counting of cells in synchronized populations, we have found that misonidazole (MIS) in clinically relevant concentrations induces cell-kinetic changes in human cells (NHIK 3025) cultivated in vitro under aerobic conditions. The effect seems to be a general lengthening of the cell cycle, affecting all phases. However, induction of this effect is phase-dependent, since only cells exposed to MIS during mitosis and/or early G1 will suffer significant cell-cycle prolongation. In exponentially growing populations this effect of MIS leads to a transient increase in the fraction of G1 cells and a corresponding decrease in the fraction of S cells. The possible significance of this effect for the clinical use of MIS is discussed.


*Br. J. Cancer* (1979) **40**, 755

### CELL-CYCLE INHIBITION BY MISONIDAZOLE OF HUMAN CELLS CULTIVATED *IN VITRO* UNDER AEROBIC CONDITIONS

T. LINDMO*, E. O. PETTERSEN† AND E. WIBE†

*From the Departments of Biophysics* and Tissue Culture†, Norsk Hydro's Institute for Cancer Research, The Norwegian Radium Hospital, Montebello, Oslo 3, Norway*

Received 27 April 1979 Accepted 20 July 1979

**Summary.—By means of flow cytometric recording of DNA histograms and counting of cells in synchronized populations, we have found that misonidazole (MIS) in clinically relevant concentrations induces cell-kinetic changes in human cells (NHIK 3025) cultivated *in vitro* under aerobic conditions. The effect seems to be a general lengthening of the cell cycle, affecting all phases. However, induction of this effect is phase-dependent, since only cells exposed to MIS during mitosis and/or early G1 will suffer significant cell-cycle prolongation.**

**In exponentially growing populations this effect of MIS leads to a transient increase in the fraction of G1 cells and a corresponding decrease in the fraction of S cells. The possible significance of this effect for the clinical use of MIS is discussed.**

MISONIDAZOLE (1-(2-nitroimidazol-1-yl)-3-methoxy-2-propanol, formerly Ro-07-0582, here abbreviated to MIS) is currently tested as an adjunct to radiotherapy in humans (Thomlinson *et al.*, 1976; Dische *et al.*, 1977). Apart from its radiosensitizing effect on hypoxic cells, MIS has been found to have a cytotoxic effect which is stronger under hypoxic than under aerobic conditions (Hall & Roizin-Towle, 1975; Moore *et al.*, 1976; Stratford, 1978; Taylor & Rauth, 1978).

While the cytotoxic effects of MIS have been studied both *in vitro* (Hall & Roizin-Towle, 1975; Moore *et al.*, 1976; Stratford & Adams, 1977) and *in vivo* (Brown, 1975), little information is so far available on explicit cell-cycle-inhibitory effects. It has been shown (Stratford & Adams, 1977; Miller & Hall, 1978) that cell growth, scored as increase in cell number with time, was almost zero in populations exposed to 5 mM MIS under aerobic conditions. Geard *et al.* (1978) have reported data on the rate of metaphase accumulation for exponentially growing Chinese hamster V79 cells in contact with MIS and inhibited in metaphase with colcemid. Accumulation was followed up to 6 h after addition of MIS, without any inhibitory effect under aerobic conditions being seen. In hypoxic cells, however, MIS slowed down progression through the cell cycle in a manner nonspecific for cell-cycle stage.

The present investigation shows that exposure to MIS under aerobic conditions has cell-cycle-inhibitory effects in human NHIK 3025 cells cultivated *in vitro*.

### MATERIALS AND METHODS

For this study exponentially growing as well as synchronized populations of the established human cell line NHIK 3025 were used (Nordbye & Oftebro, 1969; Oftebro & Nordbye, 1969). Cultivated in medium E2a (Puck *et al.*, 1957) supplied with 20% human serum and 10% horse serum, these cells have a doubling time of about 18 h (Pettersen *et al.*, 1977). The median generation time in populations synchronized by mitotic selection has been determined as 17·5–18·0 h. Corresponding durations of the various phases were: G1 6·5 h, S 8·0–8·5 h, G2+M 2·5–3·5 h

Correspondence to: T. Lindmo, Department of Biophysics, Norsk Hydro's Institute for Cancer Research, Montebello, Oslo 3, Norway.

(Pettersen *et al.*, 1977; Lindmo & Pettersen, 1979).

For the present study synchronized populations of NHIK 3025 cells were obtained by repeated mitotic selection from exponentially growing populations cultivated in 75cm$^2$ Falcon plastic flasks (Pettersen *et al.*, 1977). MIS (kindly supplied by Roche Products Ltd, U.K.), dissolved in Medium E2a to various final concentrations, was added to the cells by changing the medium either 45 min before or 2 h after mitotic selection. When MIS was added 2 h after selection (*i.e.* after the cells had attached to the bottom of the tissue culture flask) the control population received fresh, drug-free medium.

To determine the generation time of drug-treated populations, the increase in cell number was measured with an inverted phase-contrast microscope by counting the number of cells within delineated areas on the bottom of the culture flasks. The effects of MIS on the cell-cycle progression of synchronized populations were investigated by taking samples at different times for flow-cytometric measurement of DNA histograms. All the above experimental procedures were in an incubator room at 37 ± 0·2°C.

Single-cell suspensions for flow cytometric DNA measurement were obtained by trypsin treatment (0·25% trypsin, Difco, 1:250) and subsequent washing in Hanks' solution. Cells were stained for DNA measurement without previous fixation, using the DNA-specific stain mithramycin (Mithracin, Chas. Pfizer & Co. Inc.) according to the procedure by Crissman & Tobey (1974).

DNA histograms were recorded on a laboratory-built flow cytometer (Lindmo & Steen, 1977; Lindmo & Pettersen, 1979). Fluorescence from mithramycin was excited with the 457·9nm line of a 4W Argon laser and measured at wavelengths longer than 476 nm. To aid in the interpretation of registered DNA histograms, the median channel position was calculated for histograms of synchronized populations (Lindmo & Pettersen, 1979).

To investigate the inactivating effect of MIS, NHIK 3025 cells were plated in Petri dishes and exposed to various concentrations of MIS for 24 h. After rinsing, the cells were incubated in fresh, drug-free medium for 12–14 days to determine the fraction of surviving cells as expressed by their ability to form colonies.

### RESULTS

Cell survival after 24h exposure to different concentrations of MIS is shown in Fig. 1. Up to 1 mM, cell survival was about 90%, but cell inactivation became significant at higher concentrations.

The generation time for synchronized populations exposed to different concentrations of MIS from 45 min before or 2 h after mitotic selection can be estimated from the growth curves in Fig. 2. When 1mM MIS was added before selection (Panel A), the cells showed a significant cell-cycle prolongation during the first generation. This did not occur, however, when the drug was added 2 h after mitotic selection (Panel B). Thus significant cell-cycle prolongation is only induced by MIS

Fig. 1.—Surviving fractions of exponentially growing populations of NHIK 3025 cells incubated for 24 h in the presence of various concentrations of MIS. Different symbols represent independent experiments. The line is drawn between mean values at each concentration.

FIG. 2.—Relative increase in cell number as a function of time in synchronized populations of NHIK 3025 cells exposed to various concentrations of MIS from 45 *min before* (*A*) or 2 *h after* mitotic selection (*B*). Different symbols (○, △, □ and ◇) represent different concentrations of MIS (control, 1 mM, 2 mM and 4 mM respectively). These results and the flow-cytometric data in Figs 3 & 4 were obtained from independent experiments.

when the drug is present during mitosis or the beginning of Gl. Therefore, when the drug was added 2 h after mitotic selection (*i.e.* after the cells had passed the critical stage), no significant effect was induced until the cells passed through mitosis/ early Gl in the presence of the drug after the first cycle (Fig. 2B). For 2 mM MIS the results were in general agreement with the above pattern, but cell inactivation and cell loss during mitosis were no longer insignificant, and for 4 mM such effects dominated the results.

Fig. 3 shows DNA histograms of synchronized cell populations trypsinized and stained 10 h after mitotic selection. MIS was added 45 min before mitotic selection in concentrations of 0·8 and 2 mM. In the population exposed to the latter concentration, a large fraction of the cells was still in Gl 10 h after selection, while in the control population the Gl compartment was nearly depleted of cells. Cells exposed to 0·8 mM were also significantly delayed compared to the control population.

Fig. 4 shows DNA histograms of populations treated with the same concentrations of MIS as for Fig. 3, but in this case the drug was added 2 h after mitotic selection. At 19 h after mitotic selection, cell division was almost completed in the control as well as in the drug-treated populations, in agreement with the results of Fig. 2B. Thus, even the population exposed to 2 mM MIS was not significantly

FIG. 3.—DNA histograms of synchronized populations of NHIK 3025 cells trypsinized and stained 10 h after mitotic selection. Along the top are indicated the concentrations of MIS, from 45 *min before mitotic selection*. 40,000 cells were registered for each histogram. In each panel, the abscissa shows channel number in subdivisions of 10 from 0 to 120 (proportional to cellular DNA content). The ordinate shows the number of cells registered per channel in subdivisions of 1000 cells.

FIG. 4.—DNA histograms of synchronized populations of NHIK 3025 cells trypsinized and stained at indicated times after mitotic selection. Along the top are indicated the concentrations of MIS from 2 *h after mitotic selection*. Other details as in Fig. 3.

delayed by the 17h drug treatment from Gl through the rest of the first cell cycle. However, after exposure to MIS during the sensitive stage in mitosis/early Gl after the first generation, the rate of cell cycle progression was reduced. Thus, at 28 h after mitotic selection, the population exposed to 0·8 mM had not yet reached the stage represented by the control population at 25 h. Most of the cells exposed to 2 mM were still in Gl of the second generation.

### DISCUSSION

The present results show that under aerobic conditions MIS induces cell-cycle inhibition in the human cell line NHIK 3025. Without causing cell inactivation (Fig. 1) or cell loss during division (Fig. 2), a concentration of 1mM MIS leads to a significant kinetic effect which appears as a reduced rate of progression through the cell cycle. Induction of this effect occurs only at a certain stage of the cell cycle. Only cells exposed to MIS while in mitosis or in early Gl will suffer a significant reduction in cell-cycle progression rate.

A quantitative comparison of the DNA histograms in Fig. 3 with a time series of control histograms indicates that the population exposed to 0·8 mM from 45 min before mitotic selection lagged about 1 h behind the control population at 10 h. The results of Fig. 2A show that the delay at the end of the first cycle (18 h) was nearly 2 h for the lower concentration. At the end of the second generation the delay was more than 4 h, as judged from Fig. 2A. The cell-cycle prolongation induced by MIS therefore seems to be due to a reduced rate of cell-cycle progression rather than a temporary block at a certain stage of the cell cycle.

Growth curves (Fig. 2B) as well as DNA histograms (Fig. 4), which were obtained from independent experiments, demonstrated no effect of 1 mM MIS during the first generation when the drug was added 2 h after mitotic selection, but at the end of the second generation cell division was delayed about 3 h (Fig. 2B). Analysis of the flow-cytometric results (Fig. 4) indicated a 3h delay by the middle of the second cycle. These values are larger than the delay seen during the first cycle when MIS was added before mitotic selection. Thus the sensitivity to MIS in the critical period around mitosis and early G1 may be higher when the drug has been present during the preceding interphase.

It is difficult to determine whether the durations of the various phases are prolonged by the same factor as a result of exposure to MIS, *i.e.* whether the progression rate is uniformly reduced over the whole cell cycle. Analyses of series of DNA histograms complementary to those shown in Figs 3 & 4 clearly showed that G1 as well as S were prolonged after drug treatment. Owing to the short duration of G2 and mitosis, and the deterioration in synchrony with time after mitotic selection, the prolongation of G2 and mitosis was difficult to assess. However, the cell-cycle distribution of exponentially growing populations which had been exposed to MIS for the whole of one cycle was found to be similar to that of untreated control populations (see below). This finding supports the interpretation that MIS reduces the rate of progression uniformly over the cell cycle.

Geard *et al.* (1978) showed that MIS in concentrations of 5 mM had no effect on the rate of entry into mitosis of aerated exponentially growing Chinese hamster V79 cells. Their investigation considered only the first 6 h after drug administration. Effects such as those described in this report would, however, influence the rate of entry into mitosis only after a time comparable to the normal cell-cycle time. At that time cells which were just past the sensitive stage at the moment of drug addition would have reached mitosis after a normal cell cycle. The rate of entry into mitosis would then be low until the first cells to traverse the whole cell cycle at a reduced rate were entering mitosis. Thus, upon addition of MIS a gap will arise between the last cells to escape the effect in mitosis/early G1 and the first cells to traverse the cell cycle at a reduced rate. As this increasing gap is propagated through the cell cycle, transient changes in the cell-cycle distribution will occur. The first manifestation of this effect will be an increase in the G1 fraction of the cell population and a corresponding decrease in the S fraction, caused by a period of reduced rate of cell transit from G1 to S.

By flow-cytometric measurement of DNA histograms, such transient changes were demonstrated in exponentially growing populations of NHIK 3025 cells exposed to various concentrations of MIS. Compared to the normal cell-cycle distribution of 45% G1, 35% S, and 20% G2+M, the fraction of G1 cells increased by a factor of 1·4 between 6 and 12 h after addition of 2 mM MIS and there was a corresponding reduction in the fraction of S cells by a factor of 0·5. For 1 mM the differences were smaller (*i.e.* increase in G1 by a factor 1·2, and decrease in S by a factor 0·7). After 24h drug treatment, the fraction of cells in G1 and S had again resumed normal values, presumably because the whole population by then had assumed a uniformly slower proliferation rate.

The effects of MIS here presented were induced by a drug treatment which, in terms of both MIS concentration and exposure time, is relevant for clinical applications. Plasma concentrations up to 1 mM have been achieved without severe neurotoxic side effects (Kogelnik *et al.*, 1978) and the biological half-life of MIS is 10–18 h in human plasma (Dische *et al.*, 1977, 1978).

When MIS is used as a radiosensitizer of hypoxic cells in clinical radiotherapy, it may possibly induce *in vivo* cell-kinetic effects on aerobic cells comparable to those described here. Since the radiosensitivity of many cell types varies through the cell cycle, a change in the cell-cycle distribution will affect the overall radiosensitivity of the tissue. Many cell types are more resistant to radiation in G1 than in S (Sinclair, 1968). If aerobic proliferative normal tissue exhibits similar differential radio-resistance in G1, transient changes in the cell-cycle distribution as described above, if present *in vivo* after clinical administration of MIS, will lead to an increased resistance to radiation a certain time after drug addition. In principle such an effect might be utilized to reduce the radiation damage to the normal tissue within the radiation field. This would be achieved by applying the radiation when the shift in cell-cycle distribution of the normal tissue, and thus the change in its radiosensitivity, offered the largest protective effect. However, even for the most favourable case of high differential radio-resistance in G1, the increase in cell survival will be relatively small, since it will never exceed the increase in the resistant fraction of the cell population.

This work was supported by the Norwegian Cancer Society—Landsforeningen mot Kreft, of which E. Wibe is a Fellow.

### REFERENCES

Brown, J. M. (1975) Selective radiosensitization of the hypoxic cells of mouse tumors with the nitroimidazoles metronidazole and Ro-07-0582. *Radiat. Res.*, **64**, 633.

Crissman, H. & Tobey, R. A. (1974) Cell-cycle analysis in 20 minutes. *Science*, **184**, 1297.

Dische, S., Saunders, M. I. & Flockhart, I. R. (1978) The optimum regime for the administration of misonidazole and the establishment of multicentre clinical trials. *Br. J. Cancer*, **37**, Suppl. III, 318.

Dische, S., Saunders, M. I., Lee, M. E., Adams, G. E. & Flockhart, I. R. (1977) Clinical testing of the radiosensitizer Ro-07-0582: Experience with multiple doses. *Br. J. Cancer*, **35**, 567.

Geard, C. R., Povlas, S. F., Astor, M. B. & Hall, E. J. (1978) Cytological effects of 1-(2-nitro-1-imidazolyl)-3-methoxy-2-propanol (misonidazole) on hypoxic mammalian cells *in vitro*. *Cancer Res.*, **38**, 644.

Hall, E. J. & Roizin-Towle, L. (1975) Hypoxic sensitizers: Radiobiological studies at the cellular level. *Radiology*, **117**, 453.

Kogelnik, H. D., Meyer, H. J., Jentzsch, K. & 6 others (1978) Further clinical experiences of a phase I study with the hypoxic cell radiosensitizer misonidazole. *Br. J. Cancer*, **37**, Suppl. III, 281.

Lindmo, T. & Pettersen, E. O. (1979) Delay of cell cycle progression after X-irradiation of synchronized populations of human cells (NHIK 3025) in culture. *Cell Tissue Kinet.*, **12**, 43.

Lindmo, T. & Steen, H. B. (1977) Flow cytometric measurement of the polarization of fluorescence from intracellular fluorescein in mammalian cells. *Biophys. J.*, **18**, 173.

Miller, R. C. & Hall, E. J. (1978) Oncogenic transformation *in vitro* by the hypoxic cell sensitizer misonidazole. *Br. J. Cancer*, **38**, 411.

Moore, B. A., Palcic, B. & Skarsgard, L. D. (1976) Radiosensitizing and toxic effects of the 2-nitroimidazole Ro-07-0582 in hypoxic mammalian cells. *Radiat Res.*, **67**, 459.

Nordbye, K. & Oftebro, R. (1969) Establishment of four new cell strains from human uterine cervix. I. *Exp. Cell Res.*, **58**, 458.

Oftebro, R. & Nordbye, K. (1969) Establishment of four new cell strains from human uterine cervix. II. *Exp. Cell Res.*, **58**, 459.

Pettersen, E. O., Bakke, O., Lindmo, T. & Oftebro, R. (1977) Cell cycle characteristics of synchronized and asynchronous populations of human cells and effect of cooling of selected mitotic cells. *Cell Tissue Kinet.*, **10**, 511.

Puck, T. T., Cieciura, S. J. & Fisher, H. W. (1957) Clonal growth *in vitro* of human cells with fibroblastic morphology. *J. Exp. Med.*, **106**, 145.

Sinclair, W. K. (1968) Cyclic X-ray responses in mammalian cells *in vitro*. *Radiat. Res.*, **33**, 620.

Stratford, I. J. (1978) Split dose cytotoxic experiments with misonidazole. *Br. J. Cancer*, **38**, 130.

Stratford, I. J. & Adams, G. E. (1977) Effect of hyperthermia on differential cytotoxicity of a hypoxic cell radiosensitizer, Ro-07-0582, on mammalian cells *in vitro*. *Br. J. Cancer*, **35**, 307.

Taylor, Y. C. & Rauth, A. M. (1978) Differences in the toxicity and metabolism of the 2-nitroimidazole misonidazole (Ro-07-0582) in HeLa and Chinese hamster ovary cells. *Cancer Res.*, **38**, 2745.

Thomlinson, R. H., Dische, S., Gray, A. J. & Errington, L. M. (1976) Clinical testing of the radiosensitizer Ro-07-0582. II. Response of tumours. *Clin. Radiol.*, **27**, 167.